# Outcomes of Hospitalized and Critically Ill Adults with Murine Typhus, Galveston, Texas, USA, 2019–2023

**DOI:** 10.3201/eid3206.251015

**Published:** 2026-06

**Authors:** Matthew Pickich, Puneet Singh, Efstathia Polychronopoulou, Shawn P. Nishi, Lucas S. Blanton, Alexander G. Duarte

**Affiliations:** University of Texas Medical Branch, Galveston, Texas, USA

**Keywords:** murine typhus, bacteria, vector-borne infections, endemic flea-borne typhus, emerging communicable diseases, renal replacement therapy, artificial respiration, lymphohistiocytosis, hemophagocytic, Texas, United States

## Abstract

Murine typhus is a reemerging infectious disease with the potential for severe manifestations. We conducted a retrospective study to examine severe illness in those hospitalized with murine typhus in Galveston, Texas. We identified 149 cases, of which 119 (79.8%) were hospitalized and 33 (28%) required admission to the intensive care unit (ICU). ICU patients were older than non-ICU patients (54.9 vs. 47.2 years; p<0.02). Thrombocytopenia was more severe in the ICU group (101 × 10^3^ µ/L) compared to the non-ICU group (137 × 10^3^ µ/L) (p<0.01). The median time from healthcare contact in the emergency department to initiation of appropriate antibiotics was similar for the ICU group (1 day) and non-ICU group (2 days) (p = 0.26). Two deaths (1.7%) in the ICU group were attributed to multiorgan failure and hemophagocytic lymphohistiocytosis. In murine typhus–endemic regions, early recognition and prompt treatment is imperative to mitigate adverse outcomes.

Murine typhus, also known as endemic or fleaborne typhus, is caused by *Rickettsia typhi*, an obligately intracellular gram-negative organism transmitted to humans by fleas that infest rats and opossums (*1*). Murine typhus has a global distribution, and in the United States, it is endemic to South Texas, Hawaii, and southern California (*2*–*4*). Furthermore, despite its near eradication in the United States during the 1940s, murine typhus has resurged in southeastern Texas and California (*5*–*7*). Murine typhus manifests as an undifferentiated febrile illness often accompanied by headache, myalgias, nausea, and malaise. It is frequently described as a mild illness. However, severe manifestations with organ dysfunction have been reported. Those include acute kidney injury, meningoencephalitis, myocarditis, respiratory failure, and septic shock (*8*–*13*). As a consequence, persons with organ dysfunction might require treatment in the intensive care unit (ICU), especially when appropriate antimicrobial therapy is delayed or not administered (*7*,*14*). A systematic review of murine typhus infections occurring around the world among children and adults in diverse healthcare systems described organ failure and hospitalization that ranged from 2% to 86% and ICU admission in 5.9% (*15*). However, characteristics of hospitalized and critically ill patients and their outcomes after antimicrobial treatment were not well defined. We examined the clinical characteristics, treatment, and outcomes of hospitalized adults with murine typhus with organ failure leading to ICU admission in Galveston, Texas, USA.

## Methods

### Data and Cohort

We conducted a retrospective review of microbiology laboratory records at the University of Texas Medical Branch (UTMB; Galveston, TX, USA) to identify adults with typhus group rickettsiae indirect immunofluorescence assay (IFA) IgM or IgG titers of >1:128 treated during April 14, 2019–October 15, 2023. We selected those dates to include the start of available IFA data and electronic health records across our healthcare system. Although PCR amplification of *Rickettsia* spp. is a very specific testing method, clinical sensitivity of PCR from blood specimens is suboptimal and influenced by the assay, disease severity, and timing of sample collection relative to stage of illness (*16*). Amplification of cell-free DNA by next-generation sequencing also shows promise in the amplification of *R. typhi* DNA from plasma (*13*,*17*), but its cost and current turnaround time are limitations. We included symptomatic patients >18 years of age with an emergency department (ED) encounter and IFA results reactive for typhus group rickettsiae. We excluded patients <18 years of age, persons hospitalized 30 days before or after laboratory testing for rickettsioses, and incarcerated persons. We collected data from the electronic health record to determine demographics, symptoms, laboratory and radiographic results, time to diagnostic testing, antimicrobial treatment, clinical disposition, and outcomes. The Institutional Review Board at UTMB approved this study (IRB approval no. 23-0313).

### Case Definitions

We used the definitions used by the Flea-borne Typhus Case Definition/Case Classification of the Texas Department of State Health Services (*18*). In addition, we used the case classification described by the Centers for Disease Control and Prevention to categorize patients with probable or confirmed murine typhus (*19*). We defined clinical evidence as an acute febrile illness lasting <30 days with >2 of the following symptoms: headache, myalgia, rash, nausea/vomiting, thrombocytopenia, or elevated aspartate transaminase and alanine transaminase. Persons with probable murine typhus had clinical disease and supportive laboratory findings that consisted of IFA for *R. typhi* antigen taken within 60 days of illness with levels of IgG >1:128 or IgM >1:256. Persons with confirmed murine typhus had a clinically compatible illness and laboratory-confirmed results, which consisted of a 4-fold increase in *R. typhi* IgG titers by IFA between serum samples collected during the acute and convalescent phases of illness.

### Data Collection

We collected information on patient demographics, comorbidities, laboratory results, and radiographic imaging findings. An investigator (M.P.) performed all chart reviews, and a second investigator (P.S.) reviewed 60% of the charts to confirm accuracy and reliability of the abstracted data. We calculated the Charlson Comorbidity Index for each patient on the basis of clinical diagnoses (International Classification for Diseases, 10th Revision) before or on the day of IFA laboratory testing. We collected information regarding the time to laboratory diagnosis, the time from ED admission to antibiotic administration during hospitalization or within 30 days of ED encounter and admitting diagnosis leading to hospitalization. For critically ill patients, we collected data regarding source of admission to the ICU, vasopressor administration, invasive or noninvasive ventilation use, and continuous renal replacement therapy. In addition, we collected data on doxycycline and azithromycin administration during the inpatient stay or within 30 days of discharge. Both of those agents were selected for their in vitro activity to *R. typhi* (*20*). Although azithromycin is believed to be inferior to doxycycline for the treatment of murine typhus (*21*), it is sometimes used as an alternative agent (*22*) and is frequently used as part of empiric treatment for community-acquired pneumonia, a diagnosis of many persons entering the ICU.

### Data Analysis

We present patient demographic and clinical characteristics as means +SD or frequencies and percentages. We compared patient characteristics, laboratory results, and time from initial contact at UTMB to appropriate antimicrobial administration between persons admitted to the inpatient ward (noncritical care) and the ICU (critical care) using a *t*-test or nonparametric test for continuous variables as appropriate and the χ^2^ test for categorical variables. We conducted all analyses using SAS 9.4 (SAS Institute Inc., https://www.sas.com).

## Results

During April 14, 2019–October 15, 2023, we identified 149 adults in aggregate with signs/symptoms of fever, malaise, weakness, nausea, vomiting, abdominal pain, diarrhea, or some combination of those associated with typhus group rickettsial serology that met the definition for either probable or confirmed infection. Of those 149 adults, 119 (79.8%) were hospitalized; the mean age was 49.3 +16.6 years. Men represented 50.4% and women represented 49.6% of patients (Table 1). The most frequent comorbidities for hospitalized patients were obesity, diabetes, and chronic kidney disease. In the hospitalized patient group, 95 (79.8%) were classified as probable murine typhus on the basis of symptoms and IgG titers of >1:128 or IgM titers of >1:256. Twenty-four (20.1%) were classified as confirmed on the basis of symptoms and a 4-fold increase in IgG-specific titer (Table 2). Among the hospitalized group, 33 (27.7%) patients were admitted to the ICU because of hemodynamic instability, respiratory failure, delirium, or acute kidney injury. Among those critically ill patients, 27 were determined to have probable murine typhus and 6 were confirmed to have murine typhus. 

**Table 1:** Characteristics of hospitalized patients in study of outcomes of hospitalized and critically ill adults with murine typhus, Galveston, Texas, USA, 2019–2023*

Characteristic	All hospitalized patients, n = 119	Non-ICU, n = 86	ICU, n = 33	p value
Mean age, y (SD)	49.3 (16.6)	47.2 (16.4)	54.9 (15.9)	0.022
Sex				0.074
M	60 (50.4)	39 (45.35)	21 (63.6)	
F	59 (49.6)	47 (54.65)	12 (36.4)	
Race				
White	115 (96.6)	82 (95.35)	33 (100)	0.208
Non-white	4 (3.4)	4 (4.65)	0 (0)	
Ethnicity				0.258
Hispanic	43 (36.1)	32 (37.2)	11 (33.3)	
Not Hispanic	75 (63.1)	54 (62.8)	21 (63.6)	
Unknown	1 (0.8)	0 (0)	1 (3)	
Mean Charlson Comorbidity Index (SD)	1.05 (1.4)	0.98 (1.4)	1.24 (1.4)	0.247
Comorbidities				
Alcohol abuse	6 (5.0)	4 (4.7)	4 (12.1)	0.145
Renal disease	10 (8.4)	7 (8.1)	3 (9.1)	0.867
Diabetes	19 (16.0)	14 (16.3)	5 (15.1)	0.881
Cerebrovascular disease	7 (5.9)	4 (4.6)	3 (9.1)	0.357
Obesity	33 (27.7)	23 (26.7)	10 (30.3)	0.698
Most common diagnoses at time of admission (%)				
		Typhus (17.5); fever (39.5)	Fever (15.2); sepsis (33.3)	
		Sepsis (8); typhus (17.5)	Acute kidney failure (3); fever (15.2)	
		Pneumonia (4.7); sepsis (8)	Acute respiratory failure with hypoxia (3); acute kidney failure (3)	
		Altered mental status (2.3); pneumonia (4.7)	Dehydration (3); acute respiratory failure with hypoxia (3)	
		Altered mental status (2.3)	Dehydration (3)	

*Values are no. (%) except as indicated. ICU, intensive care unit.

**Table 2:** Initial murine typhus immunofluorescence titers and confirmed versus probable cases in study of outcomes of hospitalized and critically ill adults with murine typhus, Galveston, Texas, USA, 2019–2023

Category	Initial titers	Confirmed (%)	Probable (%)
IgM	1:256	4	24
	1:512	1	9
	1:1,024	11	57
IgG	1:128	2	11
	1:256	1	21
	1:512	2	8
	1:1,024	14	24
ICU		6 (18.2)	27 (81.8)
Non-ICU		18 (20.9)	68 (79.1)
Total		24 (20.1)	95 (79.8)

*ICU, intensive care unit.

The group admitted to the ICU was older and had a higher percentage of male patients than the non-ICU group. However, comorbidities did not differ significantly between the groups (Table 1). Frequent laboratory findings for hospitalized patients were elevated aspartate transaminase, alanine transaminase, and alkaline phosphatase (Table 3), but no significant difference in those initial values was noted between groups. At the time of hospital admission, impaired renal function was present in 27% (9/33) of the critically ill group, but no difference was observed in the median creatinine values of 0.99 mg/dL for the critically ill group and 0.87 mg/dL for the non-ICU group (p = 0.06) (Table 3). Leukocytosis with a leukocyte count of 12.0 + 6.0 10^3^ cells/µL was noted more often in the ICU group than in the non-ICU group (Table 3). In addition, a greater degree of thrombocytopenia (median platelet count of 97 + 59 10^3^/µL), hypoalbuminemia (median value of 3.2 + 0.6 g/dL), and elevated procalcitonin (median value of 3.8 + 4.3 ng/mL) was noted in the ICU group. As for initial chest radiographs, the ICU group was noted to have nonspecific parenchymal infiltrates and pleural effusions, but no significant differences between groups were noted.

**Table 3:** Laboratory values and radiographic findings of hospitalized adults with murine typhus, Galveston, Texas, USA, 2019–2023*

Test	No. patients tested	Non-ICU patients, n = 86	ICU patients, n = 33	Reference range	p value
Alanine aminotransferase, U/L	112	111 (68–166)	95 (70–140)	5–35	0.34
Aspartate aminotransferase, U/L	118	143 (96–216)	153 (96–224)	13–40	0.71
Alkaline phosphatase, U/L	118	140 (84–216)	137 (90–207)	34–122	0.68
Creatinine, mg/dL	119	0.87 (0.69–1.04)	0.99 (0.81–2.21)	0.5–1.4	0.06
Leukocytes, × 10^3^ cells/μL	119	7.8 (5.9–10.6)	10.8 (7.1–15.5)	4.3–11.1	0.005
Hemoglobin, g/dL	119	12.9 (11.7–14.3)	12.2 (11.3–13.6)	12.2–16.4	0.26
Platelets, × 10^3^/μL	119	117 (79–162)	97 (45–151)	135–351	0.01
Neutrophils, × 10^3^ cells/μL	117	6.0 (4.3–8.0)	8.3 (6.1–11.9)	1.99–6.95	0.002
Lymphocytes, × 10^3^ cells/μL	117	1.2 (0.84–1.96)	0.85 (0.56–1.51)	1.09–3.23 L	0.04
Monocytes, × 10^3^ cells/μL	117	0.36 (0.26–0.56)	0.38 (0.29–0.72)	0.36–1.02	0.40
Serum albumin, g/dL	118	3.5 (3.2–3.8)	3.2 (2.9–3. 5)	3.5–5 L	0.024
Serum sodium, mmol/L	119	131 (129–134)	128 (137–130)	135–145	0.022
Procalcitonin, ng/mL	44	1.1 (0.4–2.5)	3.8 (0.9–7.4)	<0.07	0.045
Chest radiograph findings, no. (%)					0.10
Clear lung fields		52 (64.2)	14 (43.8)		
Opacities/infiltrates		27 (33.3)	15 (46.8)		
Effusion		2 (2.5)	3 (9.4)		

*Values are median (interquartile range) except as indicated. ICU, intensive care unit.

The mean +SD time to receiving serologic results was similar for the critically ill and non-ICU groups: 4.4 +4.4 days for the critically ill group and 3.6 +5.8 days for the non-ICU group (p = 0.06). Diagnostic testing and drug administration were performed on the same date. All patients but 1 were treated exclusively with doxycycline, and the timing of antimicrobial therapy was similar between groups (1.6 +1.3 days for critically ill patients and 2.2 +2.1 days for non-ICU patients) (p = 0.26) (Table 4). Most patients were directly admitted from the ED to the ICU, and their deteriorating clinical course resulted in the initiation of mechanical ventilation in 5 patients and renal replacement therapy in 2 patients (Table 3). In the critically ill group, hemodynamic instability resulted in intravenous fluid resuscitation followed by vasopressor initiation in 9 patients. Hospital length-of-stay was significantly greater for the critically ill group than for the non-ICU group: 9.3 +8.4 days for the critically ill group and 4.8 +2.2 days for the non-ICU group (p<0.001) (Table 4).

**Table 4:** Comparison of treatment and outcomes of patients in study of outcomes of hospitalized and critically ill adults with murine typhus, Galveston, Texas, USA, 2019–2023*

Treatment or outcome	Non-ICU, n = 86	ICU, n = 33	p value
Mean time to diagnosis, d (SD)	3.6 (5.8)	4.1 (4.4)	0.063
Median time to diagnosis, d (IQR)	2 (1–3)	3 (1–4)	0.061
Mean time to antibiotic, d (SD)	1.6 (1.3)	2.2 (2.1)	0.264
Median time to antibiotic, d (IQR)	1 (1–2)	2 (1–3)	0.262
Doxycycline			0.145
Yes, no. (%)	82 (95.35)	29 (87.9)	
Median doxycycline inpatient duration, d (IQR)	4 (2–5)	5 (4–7)	<0.001
Azithromycin			0.127
Yes, no. (%)	11 (12.8)	8 (24.2)	
Median azithromycin inpatient duration, d (IQR)	3 (2–4)	2 (2–3)	0.489
Vasopressors, no. (%)			<0.001
Yes	5 (5.8)	12 (36.4)	
CRRT, no. (%)			0.021
Yes	0	2 (6)	
Ventilation, no. (%)			<0.001
Yes	0	5 (15.2)	
Mean hospital LOS, d (SD)	4.8 (2.2)	9.3 (8.4)	<0.001
Median hospital LOS, d (IQR)	4 (3–6)	7 (5–9)	<0.001

*CRRT, continuous renal replacement therapy; IQR, interquartile ratio; LOS, length of stay.

Two deaths occurred in the critically ill group, 1 caused by septic shock and 1 by multiorgan failure associated with hemophagocytic lymphohistiocytosis. Among the 2 deaths, 1 patient was a 66-year-old man who was admitted with shock, renal failure, and disseminated intravascular coagulation. He died within 24 hours after a cardiac arrest and exhibited laboratory features of myocarditis. The second patient, a 69-year-old man with daily alcohol consumption, was admitted with pancytopenia, ferritinemia, and elevated serum transaminase that raised concern for hemophagocytic lymphohistiocytosis, which was confirmed by bone marrow biopsy. Despite administration of doxycycline on hospital day 4 and corticosteroid therapy for hemophagocytic lymphohistiocytosis on hospital day 6, he died of septic shock and respiratory failure on day 9 of hospital admission.

## Discussion

Although murine typhus is often considered a mild illness (*23*,*24*), we identified 119 adults hospitalized for symptoms associated with murine typhus, and 28% exhibited organ failure manifesting as hemodynamic instability, acute respiratory failure, or acute kidney injury that resulted in ICU admission. Furthermore, patients with organ dysfunction admitted to the ICU were older than those not admitted to the ICU and did not have recognized immunocompromised conditions. Of note, all but 1 of the hospitalized patients were prescribed tetracycline therapy during their hospital stay, and only 1 patient received macrolide therapy. Those findings highlight the illness and complications associated with this infectious disease, as well as the challenges associated with real-time diagnosis.

Murine typhus characteristically manifests as acute febrile illness with nonspecific symptoms that can mimic those of other infectious diseases, leading to misdiagnosis and a delay in the administration of effective antimicrobial therapy. As a consequence, delays in diagnosis or treatment in elderly or immunocompromised persons can lead to complications manifesting as organ dysfunction. A systematic review of worldwide published case series reported complications in 26.1% of patients, although the authors acknowledged a reporting bias that might overestimate certain findings (*15*). Those authors described complications that primarily involved the pulmonary system as pneumonia or radiographic pulmonary infiltrates, which was noted in 128 patients and developed into acute respiratory failure in 5 persons. Other complications in order of occurrence were central nervous system involvement, acute kidney injury, and, less commonly, shock. The authors proposed that geographic variation with respect to healthcare delivery accounted for differences in the severity of illness but were unable to identify risk factors associated with organ failure. Case series of murine typhus in Texas, a recognized endemic region, have reported complications leading to hospitalization and critical care services related to acute neuropsychiatric, renal, and pulmonary involvement (*25*–*28*). A 2008 study reported hospitalization for 23 of 33 persons with murine typhus and ICU admission for 9 persons (27%) (*25*), whereas another study reviewing medical records from 2013 to 2016 described complications in 18 of 54 adults (33%), 13 of whom required ICU admission (*26*). Similarly, we identified that 28% of hospitalized persons required ICU admission; 9 (27%) required vasopressors for shock, 5 (15%) had acute respiratory failure requiring mechanical ventilation, and 2 (6%) were started on renal replacement therapy for acute kidney injury. Therefore, severe complications related to murine typhus leading to hospitalization could occur in >1 of 4 patients, manifesting as single and multiorgan failure.

Individual risk factors associated with murine typhus and organ dysfunction have been described as increasing age, alcohol abuse, and glucose 6-phosphate dehydrogenase deficiency (*8*,*27*,*28*). Although laboratory findings associated with murine typhus include elevations in hepatic transaminases, leukocytosis, hypoalbuminemia, thrombocytopenia, and hyponatremia, published data concerning laboratory findings associated with organ failure from severe murine typhus are limited (*8*,*15*). In this study, we found greater reductions in platelet count in the critically ill group than in the noncritical group at the time of initial healthcare contact. In addition, impaired renal function was more commonly observed in the critically ill group, and the mechanism for this failure has been attributed to prerenal and immune-mediated causes (*29*). Delays in appropriate antimicrobial therapy have been proposed as another risk factor for complications associated with murine typhus infection (*8*,*14*,*30*). Antimicrobial therapy resolves symptoms by halting bacterial replication (*31*), thus mitigating further endothelial damage and allowing time for host-defense mechanisms to mount an effective response (*32*). However, recovery without antimicrobial therapy has been reported, although the factors associated with that phenomenon are unclear and might relate to the quantity of inoculum, individual comorbidities, and host immune response (*15*,*26*). In this study, we identified a similar time from admission to initiation of antibiotics for both groups that suggest host-related immune response might account for the progression from mild illness to organ dysfunction to multiorgan failure. However, we are unable to accurately account for the length of time from initial symptom onset to admission, and some persons might have delayed seeking medical attention, thereby prolonging the duration of infection and encouraging the subsequent development of organ failure.

Diagnostic laboratory testing relies on serologic testing because of the similarity of murine typhus symptoms with other acute febrile illnesses, which can pose dilemmas for clinicians. In addition, although a history of flea exposure is relevant, that information is infrequently obtained and reported in <25% of patients (*15*). Serologic testing is usually conducted using IFA to detect IgG and IgM reactive to typhus group rickettsiae (commercial assays use whole-cell *R. typhi* antigen), but diagnostic limitations are associated with this approach. Furthermore, real-time reverse transcription PCR amplification of ribosomal RNA targets for *R. typhi* shows promise as a more sensitive test, but it is not yet available commercially (*13*). In addition, reactive antibodies are seldom present at detectable levels during the first few days to week of illness; therefore, acute- and convalescent-phase testing are required to soundly establish a diagnosis through seroconversion or a 4-fold increase in antibody titer. Another limitation concerns reliance on reference laboratories to perform IFA testing that can delay the test results. As a consequence, a confident laboratory diagnosis of murine typhus is retrospective in nature (*33*).

The absence of real-time testing for murine typhus can affect healthcare costs. A 2023 study examined healthcare resource utilization for persons with murine typhus and compared them to age-matched controls with influenza, which is often diagnosed with rapid point-of-care testing (*34*). Patients with influenza underwent a less expensive assessment (mean $817/patient) than did patients infected with murine typhus (mean $16,760/patient). In addition, the article reported substantially more healthcare encounters for patients with murine typhus than for patients with influenza. Thus, inability to identify and distinguish murine typhus from other febrile infections resulted in higher costs.

Although we did not examine healthcare costs, we found the mean time to diagnosis in hospitalized (noncritical) patients to be 3.6 days compared with 4.4 days in critically ill patients. Furthermore, time to initial healthcare contact and the initiation of appropriate antibiotics did not vary significantly between groups. An explanation for these findings is likely related to the early use of tetracycline-based therapy for acute febrile illnesses by clinicians, as well as infectious disease consultants who advocate for early diagnostic testing and prompt initiation of tetracycline therapy for symptomatic hospitalized patients with suspected murine typhus (*35*).

Murine typhus is associated with a low mortality rate; earlier reports have described a case-fatality rate of 0.4% and a case-fatality rate of up to 4% for hospitalized patients (*8*,*27*,*36*). In addition, a systematic review assessing mortality among 239 untreated patients with murine typhus also reported a low case-fatality rate of 0.4% (*14*). In this study, we found an associated mortality rate of 1.7% for hospitalized patients. Upon further investigation into the causes of death, hemophagocytic lymphohistiocytosis was diagnosed in 1 patient with confirmed murine typhus; despite 5 days of intravenous doxycycline and initiation of corticosteroid therapy, the patient died. Hemophagocytic lymphohistiocytosis has been described as a rare complication associated with murine typhus and is characterized by an exaggerated immune response with increased proliferation and activation of histiocytes (*37*,*38*). The development of critical illness and severe multiorgan dysfunction should prompt investigation into rickettsial infection–associated hemophagocytic lymphohistiocytosis (*39*–*41*).

The strengths of this study include the number of persons residing in an endemic area for whom clinical data were available, which enabled examination of numerous factors associated with murine typhus. The first limitation of this study is its retrospective nature, which might introduce bias. Second, our study only examined symptomatic patients seeking medical attention and might underreport the extent of disease in the community. Another limitation concerns the discrepancy between the number of confirmed infections and the number of probable infections. This discrepancy is attributable to the limitations of serologic testing; convalescent testing is seldom performed after discharge. Studies have shown a demonstrable seroprevalence of typhus group antibodies in the communities of Galveston County. Typhus group antibodies were found to be present in 1.2% of Galveston County residents in 2013 and 7.8% of residents in 2021 (*42*,*43*). Although that might raise doubt regarding the reliability of probable cases, a separate study describing the reemergence of typhus group rickettsiosis in the Galveston area indicated similarity in the signs and symptoms of confirmed and probable cases (*5*). Another limitation was the lack of clinical information regarding symptom duration before ED admission, which might have contributed to illness progression for older persons. In addition, our study did not examine for the presence of infection from spotted fever group rickettsiae. Antibodies stimulated by typhus group rickettsiae can also cross-react with spotted fever group antigen, albeit usually at titers that are at least 4-fold less than those against homologous antigen (*44*). Unless there is a history of tick bite or travel to an area with a greater abundance of ticks, spotted fever group antibodies are usually not routinely checked in conjunction with typhus group rickettsial serology at UTMB. Serum samples from Galveston residents reactive to *R. typhi* do not react with the immunodominant spotted fever group-specific outer membrane protein A (OmpA) by Western blot analysis (*42*). Although undiagnosed spotted fever group disease in our cohort is a possibility and a limitation of this study, typhus group rickettsiosis is likely the predominant cause of rickettsial illness in Galveston. The duration of onset of illness to initiation of antibiotics for our study was 1–2 days after contact at UTMB, which could be considered a limitation given that the median duration from onset of illness to initiation of doxycycline was documented to be 9 days in a previous Galveston study of cases during 2012–2016) (*45*) and 10 days in a study that examined murine typhus throughout Texas (cases during 1980–1987) (*8*). An analysis of patients admitted to a hospital close to the southern Texas–Mexico border revealed that the median time to initiate doxycycline from initial point of healthcare contact was 5 days (*26*). Although our study did not calculate the time from symptom onset to initiation of doxycycline, over time, at our institution, recognition of murine typhus seems to have led to relatively swift empiric administration of doxycycline. Finally, this study lacks data regarding antimicrobial therapy before patients sought care at the ED.

In conclusion, in symptomatic hospitalized adults with murine typhus in the Galveston, Texas, area, severe organ dysfunction manifested as shock, acute kidney injury, and acute respiratory failure in 28% of patients. Among hospitalized patients with elevated hepatic transaminases and thrombocytopenia, empiric tetracycline therapy was initiated within 1–2 days of ED admission. Risk factors associated with organ dysfunction in adults were increased age and thrombocytopenia at time of initial healthcare contact. Clinicians in endemic areas must recognize murine typhus as a potentially severe illness and institute prompt, empirical therapy with doxycycline when a case is suspected.
